# Using a web platform for equitable distribution of COVID-19 monoclonal antibodies: a case study in resource allocation

**DOI:** 10.3389/fpubh.2023.1226935

**Published:** 2023-11-28

**Authors:** Jonathon P. Leider, Sarah Lim, Debra DeBruin, Alexandra T. Waterman, Barbara Smith, Umesh Ghimire, Haley Huhtala, Zachary Zirnhelt, Ruth Lynfield, John L. Hick

**Affiliations:** ^1^Center for Public Health Systems, Division of Health Policy and Management, School of Public Health, University of Minnesota, Minneapolis, MN, United States; ^2^Minnesota Department of Health, Saint Paul, MN, United States; ^3^Center for Bioethics, University of Minnesota, Minneapolis, MN, United States; ^4^Health Sciences Technology, University of Minnesota, Minneapolis, MN, United States; ^5^Hennepin Healthcare, Minneapolis, MN, United States

**Keywords:** public health preparedness, COVID-19 response, scarce resource allocation, case study, monoclonal antibody, equitable allocation of resources, crisis standards of care

## Abstract

While medical countermeasures in COVID-19 have largely focused on vaccinations, monoclonal antibodies (mAbs) were early outpatient treatment options for COVID-positive patients. In Minnesota, a centralized access platform was developed to offer access to mAbs that linked over 31,000 patients to care during its operation. The website allowed patients, their representative, or providers to screen the patient for mAbs against Emergency Use Authorization (EUA) criteria and connect them with a treatment site if provisionally eligible. A validated clinical risk scoring system was used to prioritize patients during times of scarcity. Both an ethics and a clinical subject matter expert group advised the Minnesota Department of Health on equitable approaches to distribution across a range of situations as the pandemic evolved. This case study outlines the implementation of this online platform and clinical outcomes of its users. We assess the impact of referral for mAbs on hospitalizations and death during a period of scarcity, finding in particular that vaccination conferred a substantially larger protection against hospitalization than a referral for mAbs, but among unvaccinated users that did *not* get a referral, chances of hospitalization increased by 4.1 percentage points.

## 1 Introduction

In November 2020, the U.S. Department of Health and Human Services (HHS) notified states and territories of the impending emergency use authorization (EUA) and federal distribution of the first monoclonal antibody (mAb) outpatient therapy for COVID-19, bamlanivimab (1). Bamlanivimab and subsequent anti-SARS-CoV-2 mAbs have been found to be effective therapies to reduce the risk of progression to severe COVID-19 in high-risk patients by up to 89% (2–4). As the first U.S. Food and Drug Administration (FDA)-authorized outpatient therapy for mild-to-moderate COVID-19 (5), it was expected demand would outstrip supply during the initial rollout.

The United States has a mixed public-private payor model for health care, where federal and state governments pay some personal health care costs through public programs such as Medicare and Medicaid, while private citizens and employers also pay by way of health insurance premiums and out-of-pocket spending. During the COVID-19 response, the U.S. Congress passed legislation empowering the federal government to pay for aspects of the pandemic response. One legislative initiative empowered HHS to purchase mAbs from pharmaceutical manufacturers and then distribute to each U.S. state, territory, and federal partner. States were responsible for directing their allocations to administration sites. Thus, mAbs became central to many states’ COVID-19 response strategies. Most states used a decentralized approach, allowing hospitals or health care systems to allocate these drugs using a patchwork of methods, with mixed success in reaching patients at highest risk (6). Minnesota attempted a centralized approach, one that prioritized an ethics-oriented allocation focused on access, equity, and improved population health outcomes. There has been substantive discussion for many years regarding ethical frameworks for triage and rationing in disasters. However, there has been little movement toward applied, large-scale systems implementing these frameworks on a population level (7–10).

As the supply of mAbs was very limited when HHS began distribution (11, 12), concerns arose about scarcity and the resulting need for a process to ethically allocate mAbs among patients. Even if supply was sufficient to meet demand, a process was needed to avoid unfairly disadvantaging patients whose health systems elected not to offer mAbs or patients who were not affiliated with any health system. In Minnesota, we responded to this need through a collaboration among the Minnesota Department of Health (MDH), including its Science Advisory Team (MDH SAT), the Minnesota COVID Ethics Collaborative (MCEC), the University of Minnesota (UMN), and 116 private healthcare administration sites in the state of Minnesota. The product of this collaboration was a web-based platform -- the Minnesota Resource Allocation Platform (MNRAP) – which linked more than 31,000 Minnesotans to care during 18 months of operations and managed the allocation of resources by clinically prioritizing those most at risk for hospitalization or death, including operating a statewide lottery during deepest scarcity.

We provide here a case study in the design and implementation of the system and answer a fundamental question: how valuable was a referral from this system in the prevention of COVID-19-related hospitalization?

## 2 Context

During the COVID-19 pandemic, MDH received input from two advisory groups, MDH SAT and MCEC. MDH SAT was established in 2005 as a planning work group with the ability to provide a real-time advisory role to the MDH Commissioner during crisis response. MDH SAT has provided guidance on matters such as patient care strategies for scarce resource situations and management of pharmaceutical shortages (13). MCEC was convened in March 2020 as a statewide ethics advisory group to support COVID-19 response in Minnesota, building on contracted foundational work on the ethics of crisis response completed for the state between 2007 and 2016 (14, 15).

By early November 2020, following a series of discussions with healthcare providers across Minnesota, MDH SAT and MCEC developed initial recommendations for the MDH Commissioner to consider regarding equitable allocation of mAbs (16). These included:

- a single pathway for access to mAbs for any Minnesotan seeking care, regardless of their usual provider (or lack thereof), their ability to pay, or their immigration status
- a centralized process to assess eligibility and minimize variations in access across the state
- a mechanism to prioritize those most at risk.

The vision for the centralized allocation mechanism—the Minnesota Resource Allocation Platform (MNRAP)—was a public facing, online platform that connected eligible patients to an available appointment at the closest healthcare facility, thus alleviating the burden of searching multiple systems for appointments, and broadening access to information regarding COVID-19 therapeutics. MDH distributed mAbs to healthcare facilities within the state and issued guidance concerning the allocation of doses to patients. MDH also maintained a COVID-19 therapeutics website with educational resources for patients and providers and access to the MNRAP platform via direct link. Information was provided on clinical eligibility, the rationale for mAb treatment, and details on cost and insurance, including patients may be liable for costs of administration charged by facilities, but not the mAb itself.

On entering MNRAP, two screener options were provided, one for self-referral by a patient, and another for a friend or family member completing on behalf of a patient. The screeners included a series of questions to gauge provisional clinical eligibility for treatment based on criteria in the FDA EUAs for mAbs. Upon completion of the questionnaire, qualifying patients instantly received a referral to the location of their choosing with available capacity. They were notified by both a pop up window and an email containing the same information as the pop up. At the same time a patient received their referral, the selected location instantly received an encrypted email referral with pertinent patient information. Private healthcare entities (infusion sites) confirmed eligibility of patients, provided patient education, scheduled them, and administered the mAbs.

Within 2 months of launch, a “provider pathway” was also implemented enabling providers to refer patients quickly by completing an abbreviated questionnaire, including attestations of eligibility. The questionnaires were regularly updated as the FDA changed eligibility criteria for mAbs, issued authorization for new mAbs for treatment or new indications (e.g., post-exposure prophylaxis [PEP]), or revoked authorization for particular mAbs. Because of the need for these regular updates, the MNRAP screeners were not translated into multiple languages, though the main MDH COVID-19 therapeutics website and patient educational resources were.

On February 9, 2021, MDH launched MNRAP for use by patients, their families or friends, and providers. During the first 8 weeks of operation, 85% of all referrals were completed by providers, highlighting lack of awareness of mAbs among the public. Despite a social media campaign by MDH, numerous interviews with the press, and continued education of providers, interest in mAbs remained low until August 2021 when Minnesota faced the Delta variant. At that point, the state hired additional providers to administer mAbs to increase capacity to meet demand.

### 2.1 A centralized system to promote equity

Promoting “equitable allocation” was core to MNRAP, derived from ethical guidance instituted by MDH with input from MCEC and MDH SAT (16). The ethical guidance contained recommendations about the allocation of mAbs to patients, and thus described the standards by which MNRAP should operate. Three ethical principles grounded this ethical guidance: (1) protect the population’s health, (2) respect individuals and groups, and (3) promote fairness and equity.

We believe our system was nationally unique in providing a statewide common access point for COVID-19 therapies, as well as using a validated clinical scoring system to prioritize those seeking treatment during periods of scarcity. The advantages of our approach were:

- robust equity protections built into the system
- a centralized process to assess eligibility, provide access to mAbs even without a provider, and to minimize subjective variations in access
- prioritization of those more at risk, in contrast to the national trends at the time which saw those with fewer chronic conditions more represented among users (17)
- load balancing of supply and demand
- decreased burden on participating health systems and responsiveness to their input
- incorporation of emerging data to best promote population health outcomes
- a deliberative, collaborative, and evidence-based advisory process that provided input on ethical, clinical, and technical considerations to inform the state’s decision-making processes and allow the response to flex to changes in context.

### 2.2 MNRAP utilization

**Figure 1:**
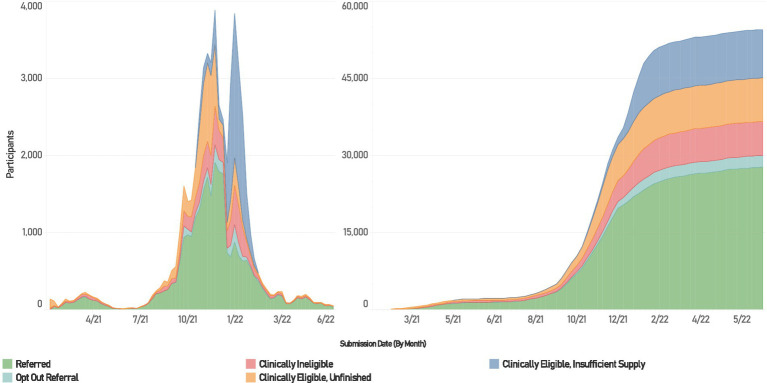
Utilization of MNRAP, February 2021 – June 2022. Left weekly participation in MNRAP. Right, cumulative participation.

**Figure 2:**
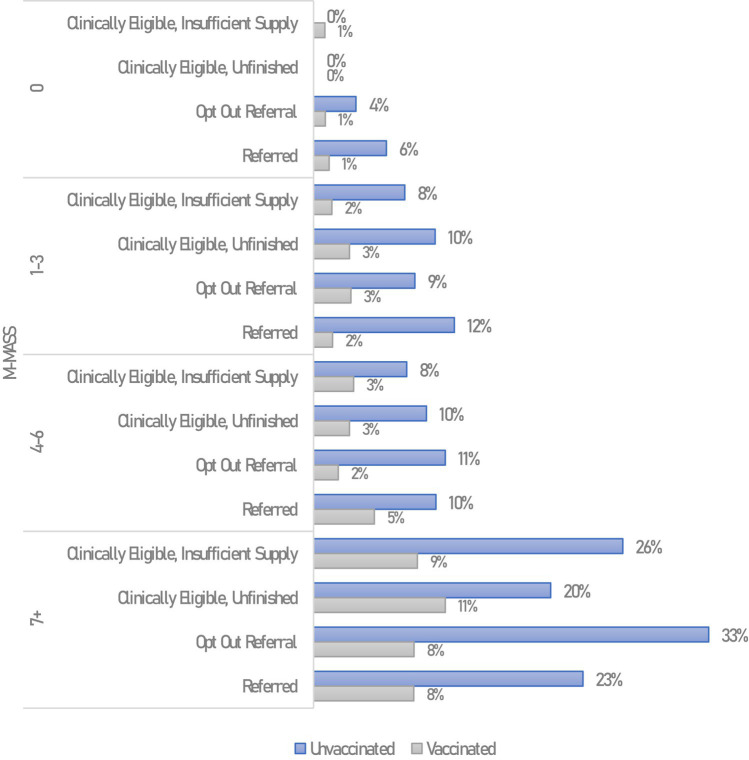
Hospitalizations among non-pregnant MNRAP participants, by MNRAP referral status, vaccination status, and modified MASS. Hospitalizations within 28 days of COVID-19 symptom onset, excludes COVID-19 reinfections.

MNRAP operated between February 9, 2021, and July 1, 2022 (Figure 1). Using the platform, 31,559 individuals received a referral for mAbs—27,066 for treatment at sites participating in the MNRAP system, 2,215 for treatment from sites opted out of (i.e., elected not to participate in) MNRAP, 2,122 for PEP for those at sites participating in MNRAP, 156 for PEP for sites opted out of MNRAP (Figure 2). PEP was available from July through December 2021 before resource constraints foreclosed availability for this indication. For this reason, presented data reflects the use of mAbs for treatment only, not PEP.

Among approximately 94,000 unduplicated individuals interacting with MNRAP, 38% dropped out of the screener (66% of whom did not fill out preliminary information on the first page, e.g., name or date of birth); 32% received referrals to participating sites and an additional 3% were referred to a site “opted out” of MNRAP; 10% were clinically eligible but did not receive a referral due to insufficient supply; 10% were clinically eligible but did not finish the survey (by confirming their chosen location or otherwise completing the screener); and 7% were clinically ineligible.

In Minnesota, although COVID-19 cases exploded and hospitals were surging beyond their capabilities, the supply of mAbs initially exceeded demand as utilization was low (18). Statewide total MNRAP referrals for mAbs crested at approximately 600/month in April 2021 and decreased to 60 in June 2021 and 120 in July 2021. Utilization then began to increase starting in August 2021 during the wave caused by the Delta variant, from 700/month to a peak of 6,650 in November 2021, leading to a paucity of available appointments, despite efforts to increase capacity, especially in the Twin Cities metropolitan area. By December 2021 Minnesota experienced significant scarcity of mAb doses, leading to the initiation of clinical prioritization followed by a lottery, to promote access for those most at risk. This was due to a surge in COVID-19 cases caused by the Omicron variant and the loss of two out of the three then-authorized mAb treatments due to lack of efficacy against Omicron (19).

### 2.3 MNRAP and scarcity

MNRAP was designed to connect eligible patients with sites offering mAbs, load-balance supply and demand across sites, and implement allocation criteria if demand outstripped supply during extended scarcity. Scarcity meant there were either not enough mAb doses (supply) to meet demand or there were not enough appointments (capacity) available despite sufficient supply of doses. When supply and capacity were sufficient to meet demand, all clinically eligible patients were referred for an appointment via MNRAP. Criteria for clinical eligibility were included in the respective FDA EUAs for mAbs.

At the start of December 2021, in response to scarcity, MDH implemented a three-stage tiered allocation system (16).

- Stage 1, the lowest level of scarcity: all provisionally eligible patients could receive referrals, although facility-level limitations in appointment capacity at times resulted in patients traveling longer distances to access mAbs.
- Stage 2, a middle level of scarcity, necessitated clinical prioritization; patients at lower risk for poor outcomes were deprioritized for referrals through MNRAP to better serve the needs of higher-risk patients. While FDA’s EUAs specified criteria for clinical eligibility for mAbs, they did not provide a basis for prioritizing patients based on risk. To prioritize patients in MNRAP, risk was determined using a modified version of the Mayo Clinic’s Monoclonal Antibody Selection Score (MASS) (20, 21) adopted by MDH as the M-MASS (16). To develop this score, MDH and its advisory groups engaged with the Mayo Clinic and the University of Minnesota to evaluate patient outcomes according to the MASS and adjust thresholds of benefit to provide mAbs to those the data supported would benefit most from treatment. In particular, pregnant women and immunocompromised patients were, at minimum, given a priority score above those who were not immunocompromised or pregnant to address their disproportionate risk. In Stage 2, MNRAP prioritized patients with a score of 1 or higher and reserved the use of mAbs for PEP for immunocompromised patients only. Following the loss of FDA authorization for bamlanivimab/etesevimab and casirivimab/imdevimab (19), the threshold score for receiving priority for referral subsequently increased to 4, and the PEP pathway was later removed. Deprioritized patients were not excluded, but only given access to appointments after higher risk patients had been referred. This occurred through active capacity tracking within MNRAP.
- Stage 3 was implemented when scarcity deepened even with Stage 2 clinical prioritization in place. During Stage 3, patients with a score of 4 or higher were entered into a weighted lottery, with patients with higher scores (representing higher risk) receiving more “chances.” Those with a score less than 4 were not entered into the lottery. Patients got a “yes” or “no” regarding allocation instantly, so lottery odds were set daily based on available supply/capacity and predicted demand. MNRAP had features to mitigate risk of poor prediction of daily demand (the number expected entrants in each clinical risk stratum), including holding in reserve a portion of each day’s supply/capacity, which was allocated through a “second chance” lottery feature at the end of each day for prioritized patients who got a” no” on their first time through the lottery or were not entered into the lottery, based on their overall risk profile (as measured by the M-MASS). Patients, as well as those entering information on behalf of patients, were notified if they were selected for referral, and were also contacted directly by the treatment site to schedule an appointment.

Patients who were clinically eligible for mAb treatment but who did not receive a referral because of scarcity, either in Stage 2 or Stage 3, were informed immediately in MNRAP as well as in an email of the challenges with supply and the need to prioritize higher risk patients. To avoid patients ‘gaming the system’ and attempting to submit multiple entries, MNRAP was designed to flag duplicates and notify the patients an entry had already been submitted with their identifying information. MNRAP also did not allow patients to stay in the system through multiple rounds of lottery draws, as it would result in more chances for treatment for those who had tested earlier in their illness and disadvantage those who tested later through no fault of their own (i.e., those without easy access to testing).

## 3 Detail and outcomes

Case data, MNRAP screening data, and mandatory reporting hospitalization data were merged to analyze outcomes associated with the use of the MNRAP screening platform. Of 49,741 unique individuals interacting with the system between February 2021 and July 2022, 43,952 were clinically eligible and 32,140 were matched on first name, last name, and date of birth against MDH COVID-19 case data (*n* = 1,214,310 reports). Importantly, matched data includes both referred patients (63%) and those who did not receive referrals (37%), thus allowing the examination of the association between MNRAP referral and hospitalization and death. Because a positive COVID-19 test was required for mAb eligibility, reasons for lack of match might be differences in spelling of one’s name, inaccuracies in the date of birth, and an increased use of at-home tests with unreported results. Methodological approaches are outlined in the technical appendix. Hospitalization and death were included as dependent variables of interest if occurring within 28 days of COVID-19 symptom onset.

### 3.1 Demographics

The mean age of MNRAP users was 53 (Appendix Table 1). Those aged 60–69 made up 20% of users, and those aged 50–59, 19.3% of users. 48% of MNRAP users were female. Overall, 89% of MNRAP users who indicated their race or ethnicity were non-Hispanic white (Table 1). The majority of referrals (12,500) came via the self-referral screener pathway, compared to 11,000 via the provider pathway and 3,000 via the friends/family pathway.

**Table 1:** Characteristics of referrals vs. non-referrals during MNRAP operations, February 2021–June 2022.

Variable	Value	Clinically eligible, insufficient	Clinically eligible, unfinished	Clinically ineligible	Opt out referral	Referred
Race/ Ethnicity	White	7,050 (79%)	2,083 (79%)	139 (69%)	1,934 (88%)	21,078 (79%)
	Black / African American	222 (3%)	75 (3%)	7 (4%)	19 (1%)	670 (3%)
	American Indian	84 (1%)	18 (1%)	4 (2%)	27 (1%)	269 (1%)
	Asian	229 (3%)	57 (2%)	9 (5%)	17 (1%)	511 (2%)
	Native Hawaiian	5 (0%)	3 (0%)	(0%)	1 (0%)	18 (0%)
	All Other	187 (2%)	56 (2%)	10 (5%)	31 (1%)	405 (2%)
	Hispanic / Latino	227 (3%)	65 (3%)	11 (5%)	42 (2%)	630 (2%)
	Prefer not to say	916 (10%)	267 (10%)	22 (11%)	138 (6%)	3,173 (12%)
Pathway	Self (I am filling out the survey)	6,593 (74%)	1,888 (63%)	2,232 (60%)	1,341 (61%)	12,556 (46%)
	Provider	1,093 (12%)	678 (23%)	606 (16%)	527 (24%)	11,453 (42%)
	Friend or family member helping fill this out	1,278 (14%)	439 (15%)	863 (23%)	347 (16%)	3,057 (11%)
M-MASS	0	3,847 (45%)	101 (14%)	88 (67%)	396 (31%)	2,763 (23%)
	1–3	3,288 (38%)	214 (29%)	23 (18%)	399 (31%)	3,096 (25%)
	4–6	1,203 (14%)	283 (39%)	20 (15%)	366 (28%)	3,965 (32%)
	7+	263 (3%)	134 (18%)	(0%)	130 (10%)	2,409 (20%)

There was a relatively similar distribution in overall referrals between clinical priority groups throughout MNRAP operations. About 9% of referrals were for pregnant people. Among non-pregnant patients, very low clinical risk (M-MASS 0) constituted 16% of referrals, low-risk (M-MASS 1–3) 23% of referrals, high-risk (M-MASS 4–6) 32% of referrals, and very high-risk (M-MASS 7+) 20% of referrals (Appendix Figures 2, 3). In the period preceding the weighted lottery (“Stage 3,” January/February 2022), non-pregnant high- and very high-risk groups constituted 43% of referrals, and pregnant people constituted 7%. During the lottery phase of MNRAP, non-pregnant high- and very high-risk groups constituted 77% of referrals, with pregnant people constituting 15% of referrals.

### 3.2 Outcomes

After excluding those with COVID reinfections (any reinfections within the analytic period, *n* = 2,402, 7.9%), 5.7% of MNRAP participants were hospitalized and 0.7% died. There was a statistically significant difference in hospitalizations by referral status (5.2% for unreferred vs. 6.1% for referred, *p* = 0.001) and a non-significant difference in deaths (0.61% for unreferred vs. 0.77% for referred, *p* = 0.122). However, M-MASS and vaccination status were positively and negatively associated, respectively, with hospitalization among non-pregnant participants and the clinical risk profile was markedly different between not referred and referred patients, with those referred having far higher risk profiles (Appendix Figures 3, 4).

Direct comparisons were drawn during the Stage 2 / 3 clinical prioritization and lottery periods among adult participants who did and did not get referrals in MNRAP (*n* = 12,128). Hospitalizations were lowest among participants in M-MASS 0 and M-MASS 1–3 groups, regardless of referral status. When considering only vaccination and clinical risk as measured by M-MASS, there *appeared* not to be an obvious referral benefit, as hospitalization was consistently lower for vaccinated participants and higher for unvaccinated participants. Those who were clinically eligible but did not receive a referral, either because they did not complete the survey or got a “no” in the lottery, did not appear to have higher frequency of hospitalization when taking into account vaccination.

Among MNRAP users matched to MDH case data, vaccination status was similarly associated with differential outcomes for deaths, by referral status. Among the very high clinical risk vaccinated MNRAP users (M-MASS 7+), 1.2% of those receiving a referral died, compared to 5.4% of very high clinical risk unvaccinated users with referrals in the same time frame (Appendix Figures 5, 6) (*p* < 0.0001).

**Figure 3:**
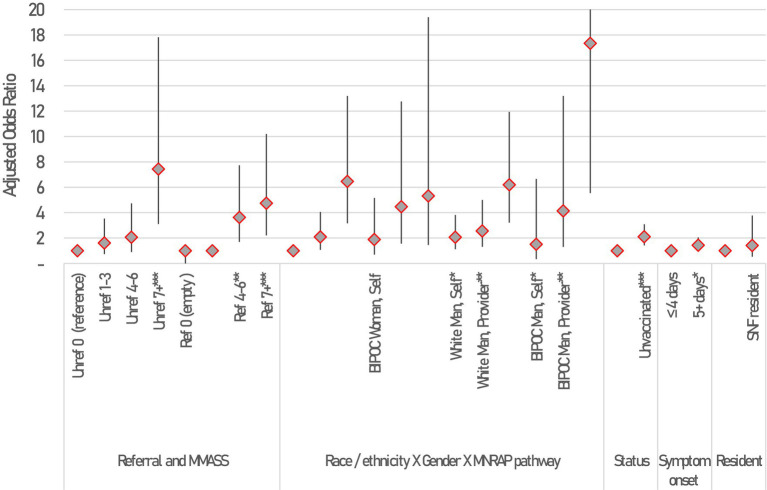
Correlates of hospitalization among MNRAP participants between January 2022 and February 2022 during mAb scarcity among adults 18+, results of a logistic model. Constant not shown. Statistical significance shown at *p < 0.05, **p < 0.01, ***p < 0.001. Error bars represent 95% Confidence intervals. Not all CIs are fully shown due to scale. 95% CIs for BIPOC Man using Friend/family pathway is [5.5, 54.2].

A logistic model was fit to better determine the relative impact of mAb referrals on hospitalizations, after accounting for clinical risk of MNRAP participants, as well as other characteristics including vaccination status, pregnancy, age, gender, race/ethnicity, days since symptom onset, whether the patient or someone else was filling out MNRAP, and whether the patient was a resident of a skilled nursing facility (SNF). White women completing the screener themselves were selected as the referent group, as they were the largest numerically. Figure 3 shows the results of a logistic model, fit during January 2022 when Minnesota operated a lottery. The lottery and its inherent randomization of persons at similar clinical risk allows for a clearer evaluation of the impact of a referral by risk on hospitalization. All else equal, the odds of hospitalization for non-pregnant adults with an M-MASS of 4–6 who did not receive a referral were twice as high as similar adults without a referral and a M-MASS of 0 (AOR 2.1, 95% CI 0.9–4.7, *p* = 0.087). The odds of hospitalization increased to 7.4 for those with M-MASS of 7+ who were unreferred (95% CI 3.1–17.8, *p* < 0.0001). For patients who did receive referrals, the odds of hospitalization for those with M-MASS 4–6 was 3.6 (95% CI 1.7–7.2, *p* < 0.001), and 4.7 for M-MASS of 7+ (95% CI 2.2–10.2, *p* < 0.0001).

We found model interactions between gender, race/ethnicity, and the type of referral through MNRAP were associated with hospital outcomes. Black, Indigenous, and People of Color (BIPOC) women whose referral was sent in by a friend or family member had increased odds of hospitalization compared to white women who completed the screener themselves (AOR 5.32, 95% CI 1.46–19.4, *p* = 0.011). Receiving a referral later in the course of one’s illness was statistically significantly associated with a higher likelihood of hospitalization (AOR 1.4 for 5+ days, 95% CI 1.01–2.05, *p* = 0.047). Finally, being unvaccinated was associated with significantly higher odds of hospitalization compared to being vaccinated (AOR 2.1, 95% CI 1.4–3.1, *p* < 0.001).

A propensity score analysis, matching participants on M-MASS, age, gender, BIPOC status, whether 5+ days since symptom onset, pathway of use, and SNF resident status, was conducted among *unvaccinated* MNRAP users without a COVID-19 reinfection during the lottery period. This analysis showed that among those clinically eligible, looking at the average treatment effect on the treated (ATET), there was an increase of 4.1 percentage points in risk of hospitalization associated with *not* being referred (95% CI 0.2–6.5%, *p* = 0.001). The mean hospitalization during this time frame was 6.8% among unvaccinated patients and 4% among vaccinated patients, though this varied substantially by M-MASS score, among other patient characteristics. Among vaccinated patients, the ATET was 0.3 percentage points and not statistically significant (95% CI, −0.1 –1.5%, *p* = 0.67), suggesting a referral may not have conferred a hospitalization benefit to the vaccinated population.

## 4 Discussion

### 4.1 On the usefulness of a MNRAP referral

While the published literature on mAb efficacy is strong (22–25), including in some subgroups such as immunocompromised patients (26, 27), our referral data are mixed. Factors such as vaccination status, age, gender, race/ethnicity, and underlying clinical risk appear to confound the relationship between a referral and hospitalization. This was particularly true during the period analyzed in this case study—the “Stage 3” weighted lottery period in January/February 2022—a period when some comparable high-risk individuals received a referral through the system and others were turned away at random. It was at this time when we might most clearly expect to see the real-world impact of a referral; instead, we saw benefits primarily to those who were unvaccinated and more at-risk, not to all groups. Our analysis is limited in MNRAP records referrals, but not whether patients received treatment; an unknown proportion of patients may have been lost to follow-up, deemed ineligible due to disease progression, or ultimately declined treatment.

An unexpected finding of this case study is vaccination conferred a substantially larger protection against hospitalization and death than a referral for mAbs from MNRAP during the entire performance period, and especially during the lottery period. The deeper propensity score analysis shows not receiving a referral was associated with a 4.1% increased risk of hospitalization in this period, all else equal, among unvaccinated users, with the benefit appearing negligible across most measures among vaccinated populations, even among those most clinically at risk. This finding is consistent with other published literature that has found a greater protective benefit to vaccination than some therapeutic agents against the risk of severe COVID-19, including sotrovimab (28). However, these studies included confirmation of adequate vaccination, such as receipt of ≥3 doses of mRNA vaccine, whereas MNRAP included only an attestation of vaccinated vs. unvaccinated.

The interactions between gender, race/ethnicity, and pathway of use in MNRAP and their impact on risk of hospitalization are illustrative of the impact of intersectionality on health disparities. Patients with lower health literacy, who may lack proficiency in English or face disadvantages in access to web-based platforms, may be more likely to have a friend, family member or provider complete the screener rather than use the self-referral pathway. These populations are also more likely to delay accessing care, to suffer from health disparities, and to have a higher burden of chronic diseases, all of which are associated with a greater risk for more severe illness and hospitalization. In addition, patients with more severe illness may have been more likely to have a friend or family member complete the screener or visit their provider who could complete the screener on their behalf.

### 4.2 Constraints and challenges

#### 4.2.1 Ethical challenges

MNRAP provided a standardized access point for scarce treatments and addressed concerns about variability in screening algorithms between healthcare systems that could lead to differential access for patients, including the potential for “hospital shopping” or gaming the system. These advantages were balanced against concerns regarding inequitable access to web-based systems to complete the screening process, and language or other factors preventing interface with the website. Differential access to transportation also presented an equity concern, particularly when the treatment site was relatively distant from the patient’s location. These challenges were mitigated by having both a family/friend assistance option and a provider pathway to support patients’ access to mAbs. To mitigate travel difficulties, a home infusion option was offered (though was not available everywhere) as well as allowing appointment transfers to closer facilities if possible. Many facilities also worked with patients to provide transport services and/or vouchers.

Another challenge was the issue of opt outs. Despite initial enthusiasm from healthcare systems for a centralized and standardized platform, some sites had concerns about integrating the platform into electronic health record pathways and whether the process of allocating equitably would result in treatment delays. These concerns were addressed in part by adapting the platform to an instant read system to minimize delay, but during the 2-month build time, some sites chose to set up their own allocation processes anyway. Ultimately, some healthcare systems chose to opt out of MNRAP, leading to the effect of creating “blind spots” in treatment allocation and the potential for inconsistency of access, and so raised concerns about inequities. About 7% of total referrals were sent to opt-out systems, who typically had significant market dominance in their geographic areas. MNRAP created a specific pathway to refer patients to opted-out systems if they indicated they were affiliated with that healthcare system or chose an opted-out system due to proximity. Opted-out systems also needed to demonstrate their screening process would perform at least as equitably as MNRAP before being allowed to opt out.

#### 4.2.2 Technical challenges

In addition to these ethical and operational challenges, several technical challenges arose:

- short turnaround time to get MNRAP built and tested changing requirements for the platform
- changing requirements for the platform
- accommodation of differing reporting capabilities at the participating clinical sites.

The UMN team built MNRAP in 2 months by leveraging existing tools and resources and utilizing considerable experience working with highly sensitive and HIPAA-protected data. A list of software used to build MNRAP and a figure of MNRAP’s database operations are included in Appendix Figure 1.

Because of the difficulty in predicting demand for mAbs, both the technical solution and the team supporting the platform needed to be flexible to respond quickly when supply, clinic availability, demand, and eligibility definitions changed. Techniques included system variables that could be adjusted at a moment’s notice that would affect the availability of “instant read” referrals, running late-day allocations for patients who were eligible but unable to get an instant read referral, and running a lottery when warranted. Federal policy changes to EUA criteria or mAb authorization occurred frequently resulting in policy changes to MNRAP, which also impacted the ability to make MNRAP screeners available in multiple languages.

Finally, a critical component of the technical workflow involved getting information from the clinical sites regarding how many appointments they were able to accept on weekdays and weekends, whether they were able to treat certain groups of patients (e.g., children or pregnant patients), whether they could perform home infusions, etc. These characteristics changed frequently, and it proved difficult for sites to provide updated information in a timely manner.

### 4.3 Limitations

Despite the benefits of MNRAP, it was not without its limitations.

- The interface required internet access and digital literacy, which has been shown to be associated with inequitable access to care (29, 30) Patients also needed an email address or telephone number as well as transportation to appointments. While some of these issues could be mitigated, for example using friends and family or home infusion where available, differential access to mAbs persisted to some extent.
- MNRAP was available in English only, which likely limited its utility for those not proficient in English. While the availability of the friend/family and provider screeners could help, not all patients may have had a trusted proxy with English language skills or healthcare provider to complete the screener. MNRAP asked all patients if a translator would be required at their appointment and passed this information to the receiving facility, but this was not a guarantee language services would be available.
- Even during a public health emergency, the state was unable to compel uniform utilization of the platform by the healthcare systems, resulting in potential inconsistency and confusion.
- Data for decision-making on those most likely to benefit from various mAbs, including real-world experience on effectiveness, were limited, particularly during the first year of MNRAP operations.
- The regular changing of authorizations and eligibility for mAbs, as well as their actual availability, likely created consternation and confusion among members of the public and the healthcare community, which translated to mixed use of MNRAP as a system.
- Outcome data took many months to clear data use agreement hurdles, cleaning, and validation to permit project analysis.
- How efficient was MNRAP relative to other systems? To our knowledge, MNRAP is the only system of its kind—but until federal/national level analyses are done, this question will remain unanswered.

## 5 Conclusion

MNRAP was created by a multidisciplinary collaborative that, over 2 years, developed a system to connect ill patients with a treatment to prevent hospitalization and death from COVID-19. Building a centralized system enabled us to provide access for patients throughout Minnesota, irrespective of their connection with a healthcare system. During scarcity, the system prioritized those most at risk for severe disease who were the most likely to benefit from treatment and provided an objective approach for allocating a scarce and potentially life-saving treatment.

Despite the limitations, MNRAP was extremely successful in matching patients to available treatments. Even with some health systems opting out of MNRAP, relatively consistent prioritization policies and strategies were adopted statewide, which were and continue to be critical to meeting ethical obligations to the community.

## Data availability statement

The data analyzed in this study is subject to the following licenses/restrictions: restricted data is not publicly available. Requests to access these datasets should be directed to leider@umn.edu - but these data are not available.

## Ethics statement

Ethical review and approval was not required for the study on human participants in accordance with the local legislation and institutional requirements. Written informed consent from the participants was not required to participate in this study in accordance with the national legislation and the institutional requirements.

## Author contributions

JL, SL, DD, AW, BS, RL, and JH: conceptualization. JL, BS, and ZZ: data curation. JL, BS, and UG: formal analysis. JL, SL, AW, and RL: funding acquisition. JL, SL, DD, and JH: investigation. JL, DD, AW, BS, and HH: methodology. SL, AW, BS, HH, RL, and JH: project administration. BS: software. SL, AW, BS, and RL: supervision. AW, BS, HH, ZZ, and JH: validation. BS, UG, and HH: visualization. JL, SL, DD, AW, BS, UG, and JH: writing – original draft. JL, SL, DD, AW, BS, UG, HH, ZZ, RL, and JH: writing – review & editing. All authors contributed to the article and approved the submitted version.
