# Supplementary material for: Using a web platform for equitable distribution of COVID-19 monoclonal antibodies: a case study in resource allocation
Source: Front Public Health. 2023 Nov 28;11:1226935. doi: 10.3389/fpubh.2023.1226935 (PMC10722896; doi:10.3389/fpubh.2023.1226935)
Supplement: Supplementary file 1 [file Table_1.docx]

Using a Web Platform for Equitable Distribution of COVID-19 Monoclonal Antibodies: A Case Study in Resource Allocation

**Jonathon P. Leider, PhD^1^, Sarah Lim, MBBCh MPH^2^, Debra DeBruin, PhD^3^, Alexandra Waterman MPH, BSN, RN^2^, Barbara Smith, MBA^4^, Umesh Ghimire, MPH, MS^1^, Haley Huhtala, BS^4^, Zachary Zirnhelt, MPH^2^, Ruth Lynfield, MD^2^, John Hick, MD^5^**

^1^ Center for Public Health Systems, Division of Health Policy and Management, University of Minnesota School of Public Health, Minneapolis MN; [ghimi022@umn.edu](mailto:ghimi022@umn.edu)

^2^ Minnesota Department of Health, St. Paul, MN; [sarah.lim@state.mn.us](mailto:sarah.lim@state.mn.us); [alexandra.waterman@state.mn.us](mailto:alexandra.waterman@state.mn.us); [Zach.Zirnhelt@state.mn.us](mailto:Zach.Zirnhelt@state.mn.us); [ruth.lynfield@state.mn.us](mailto:ruth.lynfield@state.mn.us)

^3^ Center for Bioethics, University of Minnesota, Minneapolis, MN; [debru004@umn.edu](mailto:debru004@umn.edu)

^4^ Health Sciences Technology, University of Minnesota, Minneapolis, MN; [bjs@umn.edu](mailto:bjs@umn.edu) ; [huhtala@umn.edu](mailto:huhtala@umn.edu)

^5^ Hennepin Healthcare, Minneapolis, MN; [john.hick@hcmed.org](mailto:john.hick@hcmed.org)

**Correspondence:**

[leider@umn.edu](mailto:leider@umn.edu)

**Appendix Figure 1**: MNRAP Conceptual Map


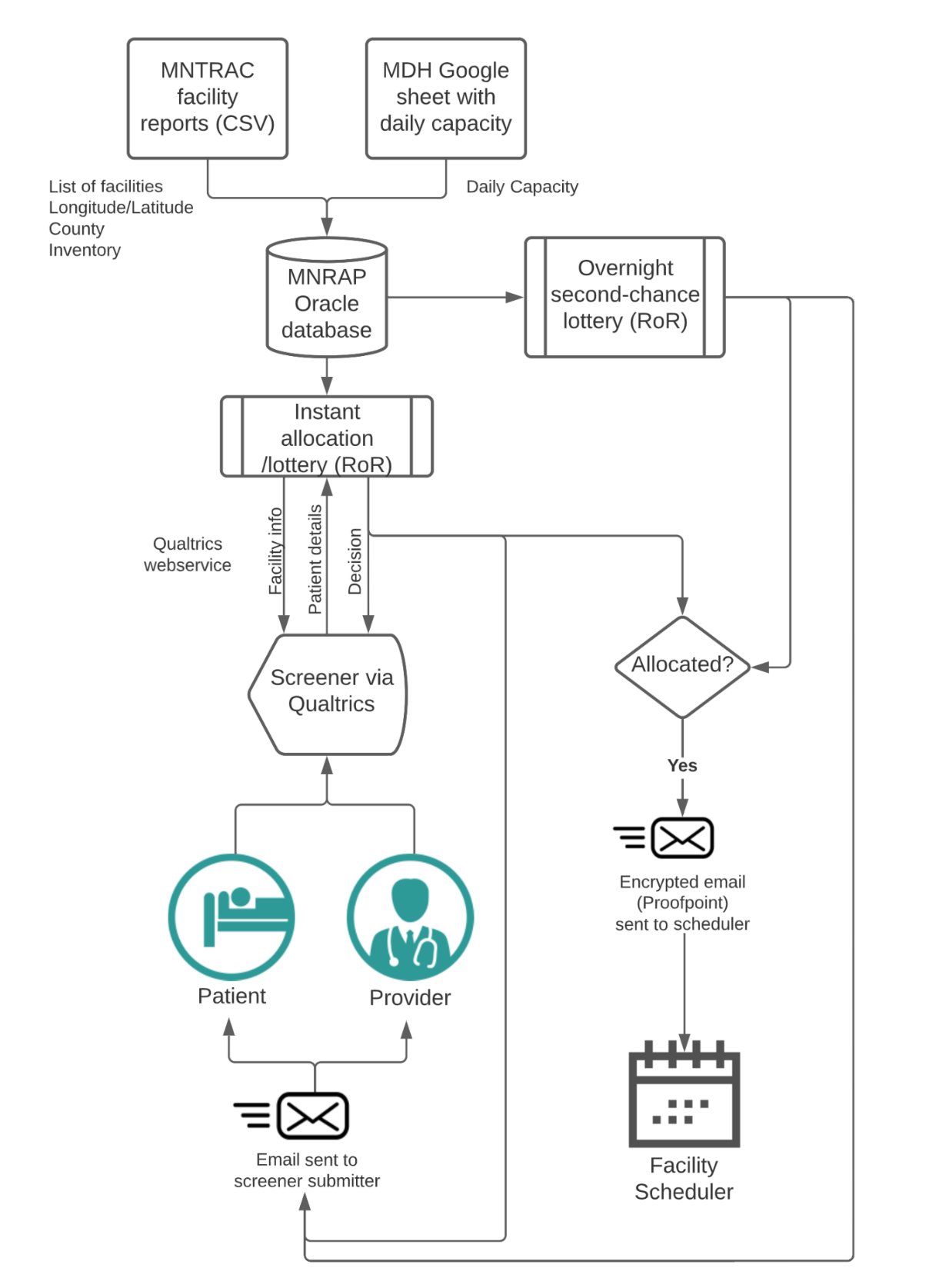


MNRAP utilizes the following technology components:

- MNTRAC is an existing tool built for and owned by the Minnesota Department of Health. It is used for healthcare systems to provide information to MDH in a secure fashion. While MNRAP was operating, the healthcare systems would enter updates to their availability and scheduler contact information into this system and then a representative from MDH would export the updates and provide a CSV to the team at UMN on a weekly basis.
- Qualtrics XM online survey tool was used to provide the screener interface that guided patients, family, friends, and providers through questions that determined eligibility and was used to hand off the collected data to the allocation logic that triggered a referral for eligible patients.
- Proofpoint secure email was used to facilitate email communications to clinical schedulers that involved PHI.
- For email communications sent to patients and providers as confirmation of their submission, a trigger was sent to the email servers owned by the Minnesota Department of Health so that the emails would come directly from MDH.
- Oracle database was used to collect data input via the Qualtrics screener and many other data points throughout the referral process.
- PHI-compliant secure servers were used to host the business logic scripts written in Ruby on Rails (RoR) which accepted data from Qualtrics and triggered the allocation resulting in referral decisions and where appropriate, triggered the referral communications.

# Appendix Figure 2: Timeline of major events in MNRAP


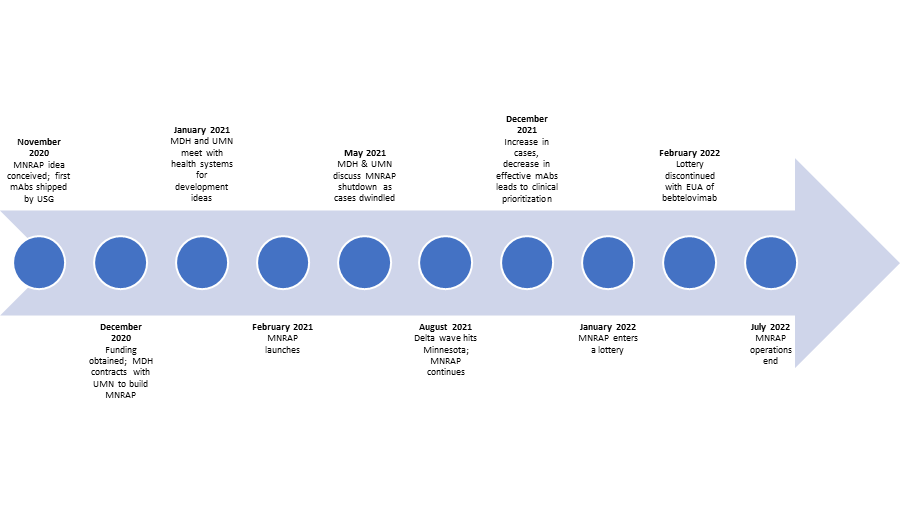


**Appendix Table 1: Characteristics of MNRAP users from February 2021 to June 2022 (and lottery period subset), including all individuals (both seeking treatment and post-exposure prophylaxis)**

| Characteristics | Lottery Period~ | Total |
| --- | --- | --- |
| **Age-categories (years)** |  |  |
| **Age (mean ±SD)** | 54 (17.9) | 53 (17.4) |
| **<30 yrs** | 8.9% | 8.5% |
| **30-39 yrs** | 15.6% | 14.9% |
| **40-49 yrs** | 15.6% | 17.1% |
| **50-59 yrs** | 17.9% | 19.3% |
| **60-69 yrs** | 21.3% | 20.4% |
| **70 yrs & above** | 20.7% | 18.5% |
| **NA** |  | 1.3% |
| Total | 8,058 | 49,740 (100.0) |
| **Gender** |  |  |
| **Woman** | 60.9% | 48.0% |
| **Man** | 39.0% | 36.4% |
| **Some other** | 0.1% | 0.1% |
| **NA** |  | 0.155 |
| **Total** | 8,058 | 49,740 (100.0) |
| Race |  |  |
| **White** | 64.5% | 69.3% |
| **Black** | 1.9% | 2.1% |
| **Am In** | 1.4% | 0.8% |
| **Asian** | 2.2% | 1.8% |
| **NH AI** | 0.0% | 0.1% |
| **Other** | 1.7% | 1.5% |
| **Latino** | 2.5% | 2.1% |
| **Prefer not** |  | 9.6% |
| **N/A** |  | 12.7% |
| Total | 6293 | 49,740 (100.0) |
| **Body mass index** | 31 (9.4) | 31 (8.8) |
| **<25 kg/m2** | 20.8% | 16.9% |
| **25-29.9 kg/m2** | 28.1% | 24.1% |
| **30-39.9 kg/m2** | 37.0% | 29.3% |
| **40 kg/m2 or higher** | 14.2% | 10.5% |
| **NA** |  | 19.1% |
| **Total** | 6744 | 49,740 (100.0) |
| **Current State** |  |  |
| **Clinically Eligible, Insufficient Supply** | 45.7% | 18.2% |
| **Clinically Eligible, Unfinished** | 2.6% | 6.7% |
| **Clinically Ineligible** | 8.3% | 7.4% |
| **Errors, Dropouts, Duplicates, and Testing** | 4.0% | 4.2% |
| **Opt Out Referral** | 3.8% | 4.8% |
| **Referred** | 35.5% | 58.7% |
| Total | 8058 | 49,740 (100.0) |
| **M-MASS** | 4 (3.2) | 2 (2.4) |
| **0** | 24.1% | 38.8% |
| **1-3** | 27.5% | 33.5% |
| **4-6** | 30.4% | 21.9% |
| **7+** | 18.0% | 5.8% |
| **Total** | 6,988 | 49,740 (100.0) |
| **Vaccinated status** |  |  |
| **Fully vaccinated** | 74.0% | 53.4% |
| **Unvaccinated or not fully vaccinated** | 26.0% | 36.0% |
| **NA** |  | 10.7% |
| **Total** | 7689 | 49,740 (100.0) |
| Symptoms |  |  |
| **Mean ±SD** | 5 (2.6) | 5 (2.8) |
|  |  |  |
| **Entrant** | 8058 | 49740 |
| **Referral** | 2862 | 29187 |

~Lottery Period started from 10 January 2022 through 13 February 2022

NA: Not Available

SD: Standard Deviation

Appendix Exhibit 2 shows the distribution of patients requesting monoclonal antibody for COVID-19 treatment. A total of 49740 participants enrolled in the MNRAP platform for mAb treatment from February 2021 to June 2022, of which 8,058 entered during the lottery period from 10 January 2022 through 13 February 2022.

The average age of MNRAP users was 54 years with most of the users belonging to the 60 to 69 (20.4%) and 50 to 59 (19.3%) age groups. The proportions of females (48%) and Whites (69.3%) were significantly high and the mean BMI of all MNRAP users was 31 (+/-SD 8.8) kg/m2. Nearly 60 percent of patients received mAb treatment through the referral pathway while 45.7 percent were clinically eligible and received the treatment during the lottery period. The average M-MASS was 2 (+/-SD 2.7) for the whole MNRAP period, whereas the mean M-MASS leveled at 4 during the lottery period. Among all MNRAP users, more than half of the participants (53.4%) were fully vaccinated. The mean number of COVID-related symptoms participants reported was 5 (+/-SD 2.8).

**Appendix Figure 2: Modified-MASS distribution of MNRAP users by age-groups**

**
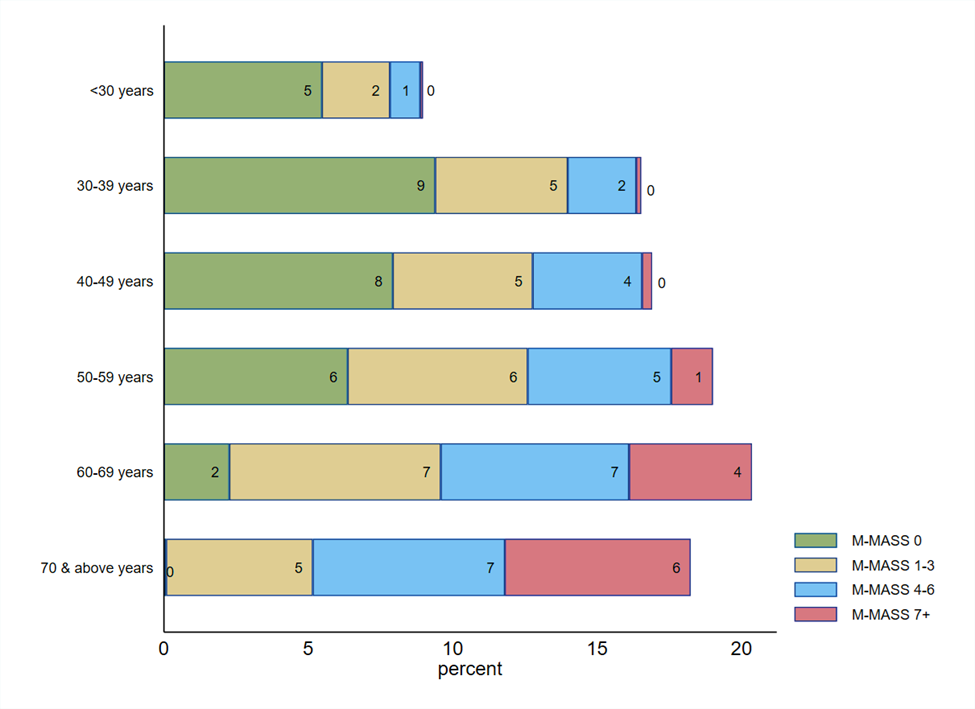
**

Among all MNRAP users, M-MASS 0 and 1 to 3 were common among younger age groups below 50 years whereas M-MASS 4 and 7+ were distinctly high among people aged 50 years and above (Appendix Exhibit 2).

**Appendix Figure 3: Distribution of body mass index and hospitalization status of MNRAP users by race category**


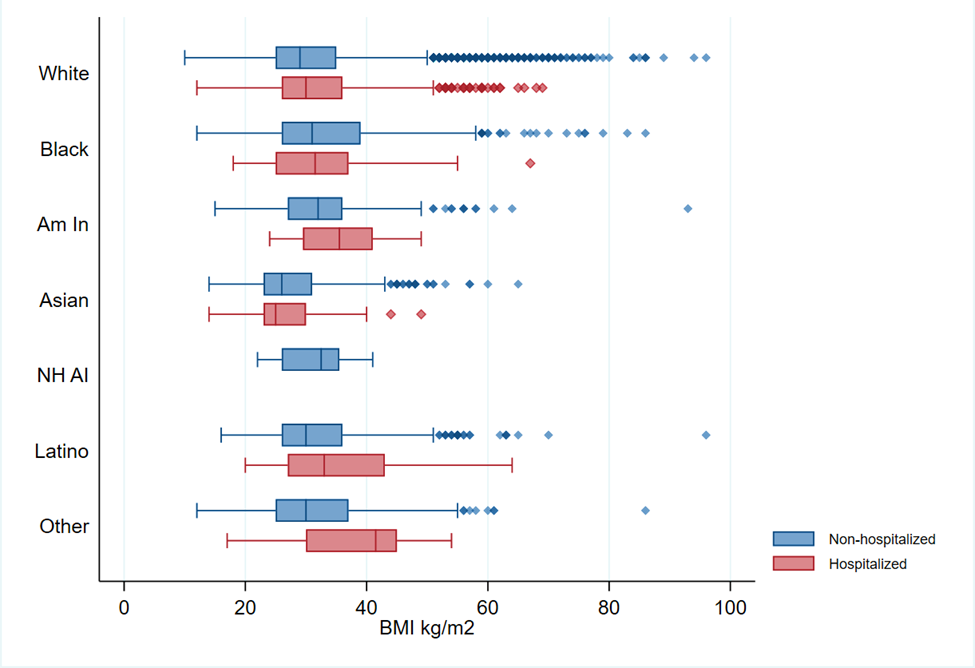


The distribution of body mass index of MNRAP users by hospitalization status varied widely across different race/ethnicity. The overall BMI of hospitalized patients was noticeably high for most of the race categories. The median BMI of hospitalized Whites, American Indians, Latino, and Other races have higher BMI. Compared to other race categories, Asians have the lowest median BMI as well as low hospitalization (Appendix Exhibit 3).

**Appendix Figure 4: Hospitalizations among pregnant people**

**
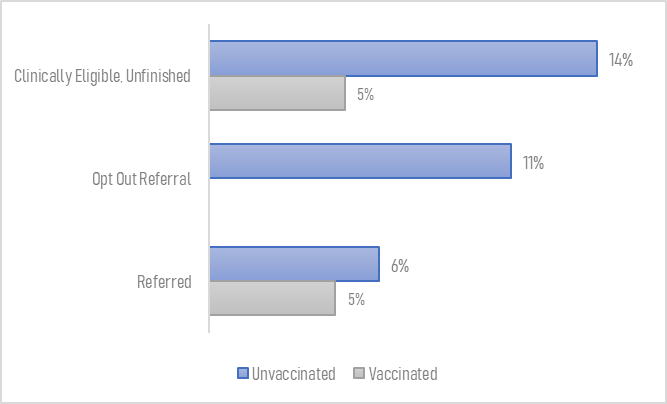
**

**Appendix Figure 5: Deaths by M-MASS during the lottery period in Minnesota**

**Technical and Methods Appendix**

**Data management**

The data used for this paper comes from the following sources:

- MNRAP dataset – Data are entered into the MNRAP screener interface by patients, family, friends, or providers. This dataset also included data points coming from the allocation scripts, including final disposition and referral information.
- MDH dataset – Data supplied to the Minnesota Department of Health by individuals, case investigators, healthcare systems for COVID test results, hospitalizations, and deaths.

For both datasets, it was necessary to identify multiple submissions for each patient. Duplicate entries were determined by matching first name, last name, and date of birth. For the MDH dataset, multiple rows per patient were flattened into one row, which holds data for up to eight entries in the original dataset. This allowed for analysis of the progression of a patient’s record, including re-infection.

For the MNRAP dataset, final disposition for each patient was categorized into one of these groups:

- Referred - patients who received a referral to a facility that was participating in the MNRAP process.
- Opt Out Referral - patients who were connected with facilities that were opted-out of the MNRAP process.
- Clinically Eligible, Insufficient Supply - patients who were clinically eligible but were not able to receive a referral due to limited capacity or limited supply.
- Clinically Eligible, Unfinished - patients who were determined to be clinically eligible but did not submit the screener once presented with the list of available facilities.
- Clinically Ineligible - patients who were determined to be not clinically eligible.
- Errors, Dropouts, Duplicates, and Testing - records that did not reach the allocation engine, either because the user terminated their screener session before submitting, the submission was determined to be a duplicate of an existing submission, or the submission was part of testing efforts.

Finally, the two datasets were merged based on matching first name, last name, and date of birth.

Hospitalization and deaths were the primary outcomes of interest. Case data from MDH, after being merged, were identified for hospitalizations and deaths, respectively, if they occurred within 28 of COVID

**Analytic approach**

Hospitalization and deaths were the primary dependent variables of interest in analysis. Bivariate comparisons were conducted using a Pearson’s chi-square. Logistic regressions were fit for the dependent variables, respectively. Independent variables included Modified Monoclonal Antibody Selection Score, gender, race/ethnicity, vaccination status, days since symptom onset, pathway of MNRAP participation (self/provider/friend or family), and whether the patient was in a skilled nursing facility. The model was fit among adults 18 and older, and excludes those who were pregnant or experienced COVID reinfection. The period of analysis was during January and early February, 2022, when the state operated the weighted lottery. The logistic model was examined for collinearity, misspecification, and goodness of fit. The c statistic for the final model was 0.75. Age and SVI were examined for inclusion but were not included in the final model given collinearity considerations. In addition to the logit, a propensity score analysis was conducted to examine the average treatment effect on the treated for those that did not receive a referral, with hospitalizations being the dependent variable of interest and lack of referrals being the primary independent variable of interest, using the same remaining covariates, using robust variance estimators. This analysis was stratified among vaccinated and unvaccinated groups during the weighted lottery period.

**List of Abbreviations:**

AOR: Adjusted odds ratio; ATET: average treatment effect on the treated; BIPOC: Black, Indigenous or people of color; BMI: Body Mass Index; CI: Confidence Interval; COVID-19: Coronavirus Disease 2019; EUA: Emergency Use Authorization; FDA: Food and Drug Administration; mAb: monoclonal antibody; MASS: Monoclonal Antibody Selection Score, MCEC: Minnesota COVID Ethics Collaborative; MDH: Minnesota Department of Health; M-MASS: Modified-Monoclonal Antibody Selection Score; MNRAP: Minnesota Resource Allocation Platform; PEP: Post-exposure Prophylaxis; SAT: Science Advisory Team; SNF: Skilled Nursing Facility; UMN: University of Minnesota; HHS: U.S. Department of Health and Human Services
